# Rapid and Low-Input Profiling of Histone Marks in Plants Using Nucleus CUT&Tag

**DOI:** 10.3389/fpls.2021.634679

**Published:** 2021-04-12

**Authors:** Weizhi Ouyang, Xiwen Zhang, Yong Peng, Qing Zhang, Zhilin Cao, Guoliang Li, Xingwang Li

**Affiliations:** ^1^National Key Laboratory of Crop Genetic Improvement, Huazhong Agricultural University, Wuhan, China; ^2^Department of Resources and Environment, Henan University of Engineering, Zhengzhou, China; ^3^Hubei Key Laboratory of Agricultural Bioinformatics and Hubei Engineering Technology Research Center of Agricultural Big Data, 3D Genomics Research Center, Huazhong Agricultural University, Wuhan, China

**Keywords:** CUT&Tag, chromatin profiling, histone modification, ChIP-seq, native nucleus, nCUT&Tag

## Abstract

Characterizing genome-wide histone posttranscriptional modifications and transcriptional factor occupancy is crucial for deciphering their biological functions. Chromatin immunoprecipitation followed by sequencing (ChIP-seq) is a powerful method for genome-wide profiling of histone modifications and transcriptional factor-binding sites. However, the current ChIP-seq experimental procedure in plants requires significant material and several days for completion. CUT&Tag is an alternative method of ChIP-seq for low-sample and single-cell epigenomic profiling using protein A-Tn5 transposase fusion proteins (PAT). In this study, we developed a nucleus CUT&Tag (nCUT&Tag) protocol based on the live-cell CUT&Tag technology. Our results indicate that nCUT&Tag could be used for histone modifications profiling in both monocot rice and dicot rapeseed using crosslinked or fresh tissues. In addition, both active and repressive histone marks such as H3K4me3 and H3K9me2 can be identified using our nCUT&Tag. More importantly, all the steps in nCUT&Tag can be finished in only 1 day, and the assay can be performed with as little as 0.01 g of plant tissue as starting materials. Therefore, our results demonstrate that nCUT&Tag is an efficient alternative strategy for plant epigenomic studies.

## Introduction

Chromatin immunoprecipitation followed by sequencing (ChIP-seq) is an efficient method for profiling histone modifications and transcription factor-binding sites (Johnson et al., 2007). In the standard ChIP-seq assay for plants (Kaufmann et al., 2010), formaldehyde-fixed nuclei are isolated and sonicated. Thereafter, the fragmented chromatin is prepared for immunoprecipitation and the ChIP DNA is purified and fragmented for sequencing library preparation. The standard plant ChIP-seq assays are complex, requiring large numbers of input cells/tissues and lasting several days from sample fixation to the sequencing-ready library. To improve chromatin profiling efficiency and save experiment time, Zhao et al. (2020) developed an enhanced ChIP-seq (eChIP-seq) protocol with modifications to the standard ChIP-seq. In eChIP-seq, the homogenate chromatin lysates are directly sonicated without nuclei purification steps. Hence, eChIP-seq considerably boosts chromatin extraction efficiency and saves a significant amount of time compared to the traditional ChIP-seq method (Zhao et al., 2020).

Recently, CUT&RUN and CUT&Tag have been developed by fusing protein A (PAT) with micrococcal nuclease and Tn5 transposase, respectively, to study chromatin state profiling using low-input samples or single live cells (Skene et al., 2018; Kaya-Okur et al., 2019). With CUT&Tag (Kaya-Okur et al., 2019), Tn5 transposase, in fusion to PAT, is tethered at specific genomic regions through the affinity of PAT to interested antibodies. Then, activation of Tn5 generates chromatin fragments for direct PCR amplification. Compared to ChIP-seq, CUT&Tag omits many steps, such as sonication, chromatin immunoprecipitation, and complicated library preparation (including DNA end repair, A-tailing, adapter ligation, and PCR enrichment). Hence, CUT&Tag enables the processing of chromatin profiling with low-input samples or even single cells and manipulation of the entire experimental procedure in only 1 day. Moreover, the PAT-based chromatin profiling strategies eliminate the requirement of the sonication and immunoprecipitation steps, enabling high-throughput identification of histone modifications at single-cell levels (Carter et al., 2019; Kaya-Okur et al., 2019; Wang et al., 2019). Most recently, Tao et al. (2020) profiled the H3K4me3 modification in cotton with high resolution and low background noise using CUT&Tag. However, the cotton CUT&Tag assays still required a significant quantity of input tissue and were time-consuming (2–3 days).

In this study, we employed our previously reported protocols for rapid and efficient nuclei isolation and developed a nucleus CUT&Tag (nCUT&Tag) protocol with protein G-Tn5 (PGT) for rapid and low-input histone modification profiling using crosslinked and fresh plant tissue. Our results showed that nCUT&Tag is an alternative strategy of ChIP-seq for fast and low-input profiling both active and repressive histone marks with crosslinked or fresh tissues from the monocots or dicots.

## Materials and Equipment

### Plant Materials, Growth Conditions, and Sample Collection

The rice cultivar from the Xian group (known as *Oryza sativa* L. ssp. *indica*), Minghui 63 (MH63), was grown in a growth chamber with the day/night cycle set at 14/10 h and a temperature of 32/28°C. The 15-day-old seedlings were collected for fresh nCUT&Tag, or crosslinked with 1% formaldehyde solution for crosslinking nCUT&Tag. A rice hybrid MHNip (MH63 × Nipponbare) was used for panicles collection. MHNip was planted in the field of Huazhong Agricultural University, Wuhan, China, and grew under normal agricultural conditions. Young panicles with 2.5-4 cm in length were collected and dual-crosslinked with 1% formaldehyde and EGS. The *Brassica napus* cultivar 2063A was grown in the growth chamber. Young leaves of 21-day-old 2063A seedlings were harvested and crosslinked with 1% formaldehyde solution.

### Regents and Equipment

1. Antibodies against proteins of interest:
Anti-H3K4me3 (Abclonal, A2357; 1 mg/ml)
Anti-H3K9me2 (Abcam, ab1220; 1 mg/ml)
2. Protein G-Tn5 fusion protein (Vazyme, cat. no. S602)
3. Phosphate-buffered saline (PBS) (Ambion, cat. no. AM9625)
4. Formaldehyde (37%; EMD Millipore, cat. no. 344198-250ML)
5. Ethylene glycol bis (succinimidyl succinate) (EGS; Thermo Fisher Scientific, cat. no. 21565)
6. Glycine (Sigma–Aldrich, cat. no. G8898-500G)
7. Sodium deoxycholate (Sigma–Aldrich, cat. no. 30970-100G)
8. Triton X-100, molecular biology grade (Promega, cat. no. H5141)
9. Tween 20 for molecular biology, viscous liquid (Sigma–Aldrich, cat. no. P9416-100ML)
10. HEPES buffer (1 M, pH 7.3, Fisher Scientific, cat. no. BP299-1)
11. NaCl solution (500 ml, 5.0M, Ambion, cat. no. AM9759)
12. Spermidine (Sigma, cat. no. S2501-1G) 2 M
13. Complete Protease Inhibitor (Roche, cat. no. 5056489001)
14. Nuclease-Free Water (1000 ml; Ambion cat. no. 4387936)
15. EDTA (pH 8.0, 0.5 M, 500 ml; Ambion, cat. no. AM9261)
16. Bovine serum albumin (BSA) (Sigma, cat. no. A1933-100G)
17. MgCl_2_ (1 M, 100 ml; Ambion, cat. no. AM9530G)
18. Sodium dodecyl sulfate (SDS, wt/vol 10%; Ambion, cat. no. AM9822)
19. Proteinase K solution (Life Technologies, cat. no. AM2548)
20. Phenol:chloroform:IAA 25:24:1 (Ambion, cat. no. AM9730)
21. GlycoBlue (Life Technologies, cat. no. AM9516)
22. Isopropanol (Sigma–Aldrich, cat. no. I-9516-500ml)
23. Sodium acetate (Ambion, cat. no. AM9740)
24. Absolute ethanol (500 ml; Sigma–Aldrich, cat. no. E7023)
25. MinElute PCR purification kit (Qiagen, cat. no. 28004)
26. TruePrep DNA Library Prep Kit V2 for Illumina (Vazyme cat. no. TD501)
27. AMPure XP beads (60 ml; Beckman, cat. no. A63881)
28. Buffer EB (250 ml; Qiagen, cat. no. 19086)
29. Dynabeads Protein G for immunoprecipitation (50 ml; Life Technologies, cat. no. 10009D)
30. Qubit 3.0 Fluorometer (Invitrogen, cat. no. Q33216)
31. Bio-Rad C1000 Thermal Cycler (Bio-Rad, cat. no. 185-1148EDU)
32. Centrifuge (Eppendorf 5810R, Swing-bucket Rotor with 15- and 50-ml Buckets, cat. no. 22628180)
33. Bioruptor Plus (UCD-300; Diagenode, cat. no. B01020001).

### Regent Setup

1. Wash Buffer (50 ml): Add 1 ml HEPES buffer (1 M, pH 7.5), 1.5 ml NaCl (5 M), and 12.5 μl spermidine (2 M) together and fill with distilled water to a final volume of 50 ml. Dissolve one tablet of Complete Protease Inhibitor in the buffer before use. Store the buffer at 4°C for up to 1 week.
2. Antibody Buffer (250 μl): Mix 1 μl EDTA (pH 8.0, 0.5 M) and 0.8 μl BSA (30%) with 250 μl Wash Buffer and chill on ice until use.
3. Transposase Incubation Buffer (50 ml): Add 1 ml HEPES buffer (1 M, pH 7.5), 3 ml NaCl (5 M), and 12.5 μl spermidine (2 M) together and bring the final volume to 50 ml with distilled water. Store the buffer at 4°C for up to 1 week. Dissolve one tablet of Complete Protease Inhibitor in the buffer before use.
4. Tagmentation Buffer (300 μl): Mix 300 μl Transposase Incubation Buffer and 3 μl MgCl_2_ (1 M) together.
5. Buffer S (500 ml): Add 25 ml HEPES buffer (1 M, pH 7.5), 15 ml NaCl (5 M), 1 ml EDTA (0.5 M), 5 ml Triton X-100, 5 ml sodium deoxycholate (10%), and 50 ml SDS (10%) together; mix the solution well and bring the final volume to 500 ml with distilled water. Sterile filtrate and store at room temperature for up to 6 months.
6. Buffer F (500 ml): Add 25 ml HEPES buffer (1 M, pH 7.5), 15 ml NaCl (5 M), 1 ml EDTA (0.5 M), 5 ml Triton X-100, and 5 ml sodium deoxycholate (10%) together; mix the solution well and bring the final volume to 500 ml with distilled water. Sterile filtrate and store at 4°C for up to 6 months.
7. Binding Buffer (10 ml): Add 200 μl HEPES buffer (1 M, pH 7.5), 100 μl KCl (1 M), 10 μl CaCl_2_ (1 M), 10 μl MnCl_2_ (1 M) together and bring the final volume to 10 ml with distilled water. Store at 4°C for up to 6 months.

## Methods

### Nuclei Isolation

**FIGURE 1:**
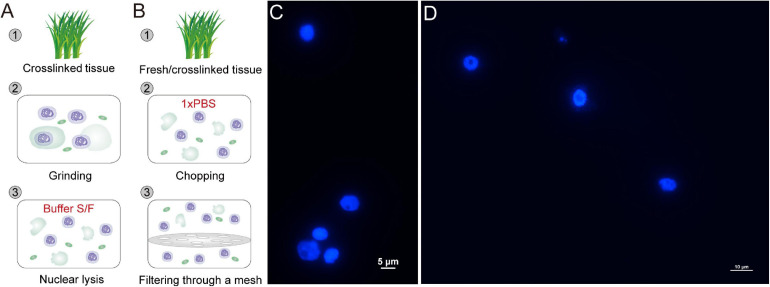
Rapid and efficient isolation of high-integrity nuclei. **(A,B)** Two strategies for nuclei isolation with crosslinked or fresh tissue. Crosslinked tissue is ground to fine powder and lyzed with Buffer S and Buffer F **(A)**. Crosslinked or fresh tissue is chopped to homogenate lysates in PBS and filtered through a mesh **(B)**. The released nuclei are collected by centrifugation. The nuclei isolated with Buffer S/F **(C)** or PBS **(D)** are stained with DAPI and observed under a fluorescence microscope.

Formaldehyde-fixed nuclei are isolated according to our previously reported protocols (Figure 1A) (Zhao et al., 2020). Briefly, 0.1 or 0.01 g of crosslinked tissue is ground to fine powders in liquid nitrogen. The powder is suspended with 300 μl Buffer S and lyzed at 4°C for 30 min with rotation. Then the 300 μl lysates are mixed with 1.2 ml Buffer S and lyzed at 4°C for 15 min with rotation. Finally, the homogenate lysates are centrifuged at 1000 *g* for 10 min at 4°C, and the nuclei are collected.

The native nuclei from fresh tissue, as well as formaldehyde-fixed nuclei from crosslinked tissue, can be isolated following a simple and fast strategy (Figure 1B) (Sun et al., 2020). The plant tissue is chopped thoroughly to complete homogeneity in a plastic petri dish with 1 ml 1 × PBS (containing protease inhibitor) on ice. The homogenate is filtered twice through a layer of Miracloth. The nuclei are isolated by centrifuging the filtrate in a swinging bucket rotor at 1000 *g* for 10 min at 4°C.

**FIGURE 2:**
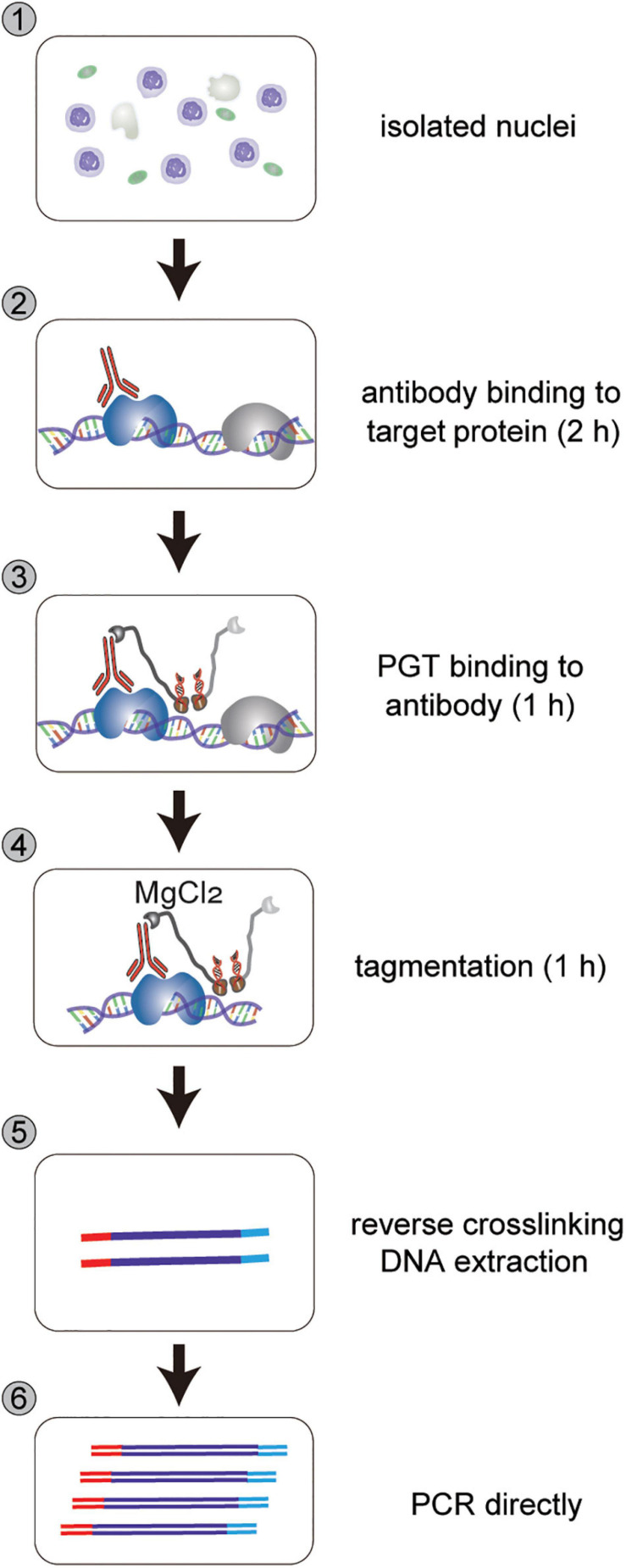
Workflow of nCUT&Tag. nCUT&Tag starts with isolated nuclei, followed by antibody binding to target protein for 2 h, PGT binding to antibody for 1 h, tagmentation for 1 h, reverse crosslinking, and direct PCR for library DNA enrichment. For fresh tissue, reverse crosslinking (step 5) can be omitted. Tagmentation DNA is purified directly using a DNA Purification kit.

The collected nuclei are stained with DAPI and observed under a fluorescence microscope. All eChIP-seq libraries are prepared following our reported protocols with Buffer S/F isolated nuclei (Zhao et al., 2020). nCUT&Tag starts with fixed or native nuclei, followed by subsequent antibody binding to proteins of interest, PGT binding to antibodies, tagmentation, DNA purification, library preparation, and sequencing (Figure 2). The following procedures are a detailed introduction of the nCUT&Tag protocol.

### Procedures for nCUT&Tag

#### Antibody Binding to Target Protein

1. Wash the nuclei pellet twice with 500 μl ice-cold Wash Buffer. Centrifuge in a swinging bucket rotor at 600 *g* for 3 min at 4°C; discard Wash Buffer.
2. Resuspend the nuclei pellet in 200 μl ice-cold Antibody Buffer. Divide into two 1.5 ml tubes with 100 μl each.
3. Add 1–5 μg antibody and IgG to the two 100 μl suspensions, respectively.
4. Incubate at 4°C for 2 h with rotation.

#### PGT Binding to Antibody

1. Centrifuge in a swinging bucket rotor at 600 *g* for 3 min at 4°C. Discard the Antibody Buffer.
2. Wash the nuclei pellet with 800 μl ice-cold Wash Buffer. Centrifuge in a swinging bucket rotor at 600 *g* for 3 min at 4°C; discard Wash Buffer.
3. Repeat Step 2 twice.
4. Mix 100 μl Transposase Incubation Buffer and 0.58 μl assembled PGT (final concentrate: 0.04 μM). Resuspend the nuclei pellet in the 100 μl transposase mixture with gentle vortexing.
5. Incubate at 4°C for 1 h with rotation.

#### Tagmentation

1. Centrifuge in a swinging bucket rotor at 600 *g* for 3 min at 4°C. Discard the supernatant.
2. Wash the nuclei pellet with 800 μl ice-cold Transposase Incubation Buffer. Centrifuge in a swinging bucket rotor at 600 *g* for 3 min at 4°C; discard Transposase Incubation Buffer.
3. Repeat Step 2 twice.
4. Mix 300 μl Transposase Incubation Buffer and 3 μl MgCl_2_ together and resuspend the nuclei pellet.
5. Incubate at 37°C for 1 h.

#### DNA Purification

1. Add 10 μl EDTA (0.5 M) and 3 μl SDS (10% wt/vol) to stop tagmentation.
Note: for fresh tissue, the Qiagen MinElute PCR purification kit (Qiagen, cat. no. 28004) is optional for DNA purification without prior reverse crosslinking. It saves much time.
2. Add 2.5 μl proteinase K solution and incubate at 50°C for 1 h to release DNA.
3. Add an equal volume of phenol–chloroform–isoamyl alcohol (pH 7.9) to the tagmentation product and mix vigorously.
4. Spin MaXtract High Density tubes at 16,000 *g* for 2 min at room temperature. Transfer the mixture in Step 3 to the centrifuged MaXtract High Density tubes and centrifuge at 16,000 *g*, at room temperature for 5 min.
5. Transfer upper aqueous phase above the gel matrix to fresh 1.5-ml tubes; add 30 μl 3 M sodium acetate (pH 5.5), 2 μl GlycoBlue, and 330 μl isopropanol and mix them well.
6. Incubate and cool down at −80°C for 30 min.
7. Centrifuge at 16,000 *g* for 20 min at 4°C.
8. Wash the pellet twice with 1 ml 75% ethanol.
9. Air-dry the DNA pellet and dissolve the DNA with 50 μl QIAGEN Buffer EB.
10. Quantitate DNA using Qubit3.0 according to the manufacturer’s instructions.

#### PCR Enrichment, Library DNA Purification, and Sequencing

50–100 ng PGT cut DNA is used for direct PCR enrichment according to the TruePrep DNA Library Prep Kit manual (Vazyme, cat. no. TD501). The PCR is performed for 13–15 cycles. PCR enriched library DNA is purified and size-selected with AMPure XP beads, and sequenced with pair-end 150 at the Illumina HiSeq2500 or HiSeq X Ten sequencing platforms.

### Procedures for Low-Input nCUT&Tag

Collect 0.1 or 0.01 g of crosslinked tissue and grind to fine powders in liquid nitrogen. Resuspend the powder with 300 μl Buffer S and lyze at 4°C for 30 min with rotation. Mix the 300 μl lysates with 1.2 ml Buffer S and lyze at 4°C for another 15 min with rotation. Centrifuge the homogenate lysates at 1000 *g* for 10 min at 4°C and collect the nuclei.

#### Binding Nuclei to Concanavalin A-Coated Magnetic Beads (Con-A Beads)

1. Wash the nuclei pellet twice with 500 μl ice-cold Wash Buffer. Centrifuge in a swinging bucket rotor at 600 *g* for 3 min at 4°C; discard Wash Buffer.
2. Wash 20 μl Con-A beads with 500 μl Binding Buffer twice to activate Con-A beads. Place the tube on a magnet stand and remove the liquid.
3. Resuspend Con-A beads with 100 μl Binding Buffer. Add the activated beads to isolated nuclei and incubate the mixture at 4°C for 15 min.

#### Antibody Binding to Target Protein

1. Discard the liquid and collect nuclei by a magnetic stand.
2. Resuspend the nuclei in 200 μl ice-cold Antibody Buffer. Divide into two 1.5 ml tubes with 100 μl each.
3. Add 1 μg antibody and IgG to the two 100 μl suspensions, respectively.
4. Incubate at 4°C for 2 h with rotation.

#### PGT Binding to Antibody

1. Discard the liquid and collect nuclei by a magnetic stand.
2. Wash the nuclei with 800 μl ice-cold Wash Buffer. Discard the liquid and collect nuclei by a magnetic stand.
3. Repeat Step 2 twice.
4. Mix 100 μl Transposase Incubation Buffer and 0.58 μl assembled PGT (final concentrate: 0.04 μM). Resuspend the nuclei in the 100 μl transposase mixture with gentle vortexing.
5. Incubate at 4°C for 1 h with rotation.

#### Tagmentation

1. Discard the liquid and collect nuclei by a magnetic stand.
2. Wash the nuclei with 800 μl ice-cold Transposase Incubation Buffer. Discard the liquid and collect nuclei by a magnetic stand.
3. Repeat Step 2 twice.
4. Mix 300 μl Transposase Incubation Buffer and 3 μl MgCl2 together and resuspend the nuclei.
5. Incubate at 37°C for 1 h.

#### DNA Purification and Library Preparation

Purify the tagmented DNA and prepare the sequencing library following the procedures as described in the nCUT&Tag protocol above.

### Bioinformatic Analysis

Trimmomatic (v0.32) (Bolger et al., 2014) is used to remove low-quality reads and to trim low-quality bases as well as adapters, with the following parameters: “ILLUMINACLIP:/adapters/TruSeq3-PE.fa:2:30:10:8:True SLIDINGWINDOW:4:15 MINLEN:50 HEADCROP:10 LEADING:5 TRAILING:5.” Trimmed reads are aligned to the MH63 reference genome (MHRS2) (Zhang et al., 2016) or *B. napus* reference genome (Chalhoub et al., 2014) using BWA (v0.7.17) mem with default settings (Li and Durbin, 2009). Then alignments with MAPQ < 30 and duplicated reads are discarded using samtools (v1.9) (Li et al., 2009). Peak calling for H3K4me3 uses macs2 (v2.1.1) with the following parameters: macs2 callpeak -t treat_bam -c control_bam -f BAMPE -B -q 0.05 -g 3.6e + 8 (-g 1.1e + 9 for *B. napus*) (Zhang et al., 2008). Broad peak calling for H3K9me2 is similar to the narrow H3K4me3 peak calling with an additional parameter –broad. Scatterplots, correlation plots, and the signal heatmaps are created using deepTools (v2.5.3) (Ramirez et al., 2014) as previously described (Zhao et al., 2020). Annotation of peaks is performed using homer (v4.11) annotatePeaks.pl with default parameters (Heinz et al., 2010). To compare the robust profiles of nCUT&Tag and eChIP-seq, we randomly extracted 500-K, 1-M, 2-M, 4-M, 8-M, 16-M, and 24-M valid clean reads from each samples to call peaks and calculate fraction of reads in peaks (FRiP) values as described in Kaya-Okur et al. (2019).

## Results

### Rapid and Efficient Isolation of High-Quality Nuclei

CUT&RUN and CUT&Tag were initially developed with human live cells (Skene et al., 2018; Kaya-Okur et al., 2019). With digitonin treatment, the membrane of cell and nucleus was permeabilized so that antibody and PAT/PGT can spread into the nuclei without compromising nuclear integrity. Since cell walls are present in plant cells, it is difficult for antibody and PGT to penetrate the cells and nuclei. As an alternative, the previously reported CUT&RUN and CUT&Tag in plants started with isolated nuclei rather than live cells (Zheng and Gehring, 2019; Tao et al., 2020). However, the nuclei isolation protocols require significant material and much time for completion because of the multiple purification steps. Here, we employed two simple protocols for fast nuclei isolation with formaldehyde-fixed tissue or fresh tissue (Figures 1A,B). For the Buffer S/F method (Figure 1A), formaldehyde-fixed tissue is ground into fine powder in liquid nitrogen and lyzed with Buffer S and F. The released nuclei are collected by centrifuged at 1000 *g* for 10 min at 4°C. In the PBS strategy (Figure 1B), fixed or fresh tissue is chopped to complete homogeneity in a plastic petri dish with 1 × PBS (containing protease inhibitor) on ice and filtered twice through a layer of Miracloth. The nuclei are isolated by centrifuging the filtrate in a swinging bucket rotor at 1000 *g* for 10 min at 4°C. All the procedures can be finished within tens of minutes. Both the two strategies isolate high-integrity nuclei (Figures 1C,D). It is worth noting that the PBS strategy is a mild and fast method for nuclei isolation. It can be used for isolating nuclei from both cryopreserved crosslinked tissues and fresh tissues, while the Buffer S/F, which contains high-concentrate SDS, is a relatively harsh strategy that may be not suitable for fresh tissues. However, compared to the PBS strategy, which may lost too much nuclei (more than 80%) during the mesh-filtering step, the Buffer S/F method is a better choice for isolating high-yield and high-quality nuclei from low-input crosslinked tissues (Zhao et al., 2020).

### nCUT&Tag for Rapid Chromatin Profiling With Crosslinked Tissue

The isolated nuclei were then directly incubated with antibodies and subsequently with PGT fusion protein (Figure 2). The PGT tagmentation reaction was activated by adding divalent magnesium ions to the incubated nuclei, and DNA fragmentation reactions occurred around the histone modification sites. Finally, the fragmented DNA was purified for sequencing library preparation.

**FIGURE 3:**
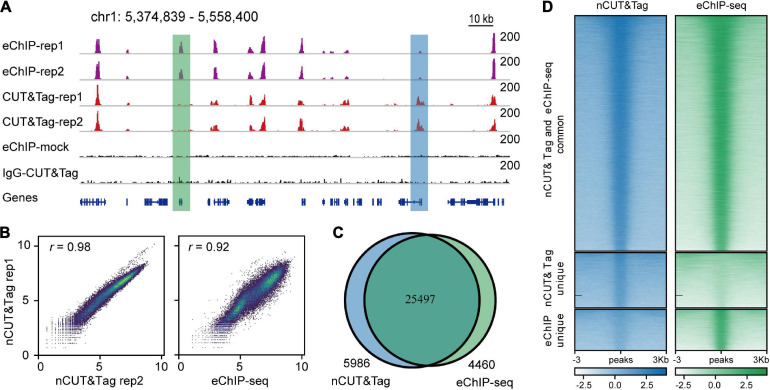
nCUT&Tag for fast chromatin profiling with crosslinked tissue. **(A)** Representative H3K4me3 landscapes across chr1:5,374,839–5,558,400 of the rice genome generated by eChIP-seq and nCUT&Tag. The green and blue boxes show peaks detected only by eChIP-seq and nCUT&Tag, respectively. **(B)** Scatter plots showing the Spearman’s correlations for the two H3K4me3 nCUT&Tag replicates (left), and between the nCUT&Tag and eChIP-seq data (right). **(C)** Venn diagram showing the overlap of H3K4me3 peaks detected by eChIP-seq and nCUT&Tag. **(D)** Comparison of the H3K4me3 eChIP-seq and nCUT&Tag signals.

Using nCUT&Tag, we first profiled the active chromatin features with H3K4me3 antibody using formaldehyde-fixed rice young panicles (Figure 3A). We performed two biological replicates of nCUT&Tag with ∼1 g of finely ground panicle powder. The nuclei were released by adding buffer S and buffer F (Zhao et al., 2020). The homogenate lysates were then centrifuged for 3 min; the nuclei pellets were used to conduct nCUT&Tag. The two replicates showed a high degree of reproducibility (*r* = 0.98, Spearman’s correlation) and a high correlation with the H3K4me3 eChIP-seq data (*r* = 0.92, Spearman’s correlation) (Figure 3B and Supplementary Figure 1A). The two nCUT&Tag replicates totally identified 31,483 high-confidence H3K4me3 peaks in rice young panicles (31,436 and 27,857, respectively) (Supplementary Table 1); among the 31,483 peaks, approximately 80% (25,497 peaks) were also detected by the eChIP-seq experiments (Figures 3C,D). Significantly, 5986 peaks were detected by nCUT&Tag only, while 4460 peaks were detected by eChIP-seq only (Figures 3A,C), indicating that the two different strategies might have distinct advantages in detecting specific histone modification sites.

The H3K4me3 peaks mainly enriched around the transcription start sites (TSS) (Supplementary Figure 3A), consistent with our previous eChIP-seq data (Zhao et al., 2020). In addition, peak annotation showed that more than 50% H3K4me3 peaks were distributed at gene promoters, the first exons, and the first introns; about 20% were distributed at transcription end sites (TES) and intergenic regions; the remainders were distributed across other exons and other introns (Supplementary Table 2). The distribution profiles of nCUT&Tag peaks showed high consistency with that of eChIP-seq peaks (Supplementary Figure 4).

Enhanced ChIP-seq is an efficient protocol in profiling histone marks. It was used to map rice and *B. napus* reference epigenomes with considerably low background noise (Zhang et al., 2020; Zhao et al., 2020). To compare the signal-to-noise ratio of nCUT&Tag relative to eChIP-seq, we downsampled the sequencing reads at varying depth from 1-M to 24-M. Then we called peaks and calculated FRiP values under the same sequencing depth (Supplementary Table 3). The results showed that eChIP-seq data exhibited higher signal-to-noise ratio than nCUT&Tag. However, using the 8-M nCUT&Tag reads, we called 27,043 peaks, which were nearly as much as that from 16-M eChIP-seq reads (27,659 peaks) (Supplementary Table 3). Our results indicated that nCUT&Tag showed a little bit higher background noise than the eChIP-seq protocol, but nCUT&Tag detected comparable peaks with much less sequencing reads.

### nCUT&Tag for Profiling Both Active and Repressive Histone Marks With Fresh Tissue

Furthermore, we conducted H3K4me3 nCUT&Tag for native nuclear chromatin profiling with fresh rice seedlings. We isolated native nuclei, according to Sun et al. (2020). A few pieces of young leaves were chopped into homogenate lysates in PBS buffer. The lysates were filtered twice through a mesh; nuclei were collected by centrifugation and used to perform nCUT&Tag. After stopping the tagmentation reaction, the fragmented DNA was directly purified following the procedure reported for ATAC-seq (Buenrostro et al., 2013; Sun et al., 2020) using a Qiagen MinElute kit (QIAGEN, cat. no. 28004) that eliminates the reverse-crosslinking steps and is a rapid DNA purification protocol.

**FIGURE 4:**
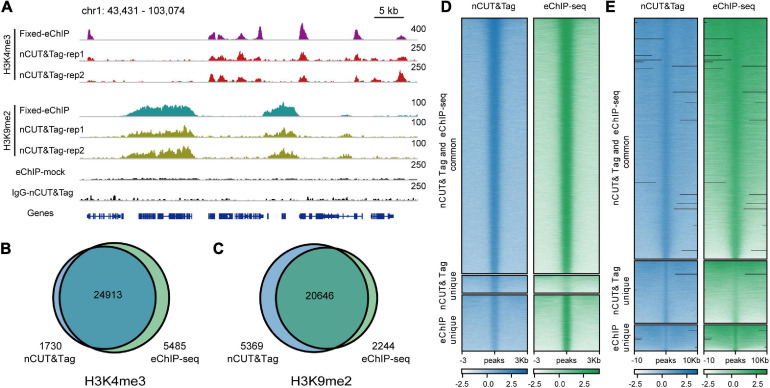
nCUT&Tag for chromatin landscape profiling with non-crosslinked tissue. **(A)** Genome browser screenshot showing H3K4me3 and H3K9me2 nCUT&Tag data for fresh rice seedlings. The H3K4me3 and H3K9me2 eChIP-seq data were generated with crosslinked seedlings. **(B)** Venn diagram showing the overlap of H3K4me3 peaks detected by fresh nCUT&Tag and crosslinked eChIP-seq. **(C)** Venn diagram showing the overlap of H3K9me2 peaks detected by fresh nCUT&Tag and crosslinked eChIP-seq. Comparison of nCUT&Tag and eChIP-seq signals for H3K4me3 **(D)** and H3K9me2 **(E)**.

The fresh nCUT&Tag showed a high correlation with the fixed H3K4me3 eChIP-seq data (*r* = 0.92, Spearman’s correlation) (Supplementary Figures 1B, 2A). The two replicates totally called 26,543 peaks (21,203 and 23,545, respectively, Supplementary Figure 4A and Supplementary Table 1). Among the 26,543 peaks, 24,913 (93.86%) were also detected by eChIP-seq. Strikingly, 1730 peaks were detected by fresh nCUT&Tag only, while 5485 peaks were detected by fixed eChIP-seq only (Figures 4B,D). In fact, there were slight signal enrichment in nCUT&Tag libraries at the 5485 eChIP-seq unique peak regions (Figure 4D). A possible explanation for that many peaks were only detected by eChIP-seq may be the lower sequencing depth of the nCUT&Tag libraries relative to the eChIP-seq data (Supplementary Table 1). The fresh nCUT&Tag signal showed similar enrichment as that of crosslinking eChIP-seq, mainly around the TSS (Supplementary Figure 3B). The fresh nCUT&Tag peak distribution profiles were also similar to that of crosslinking eChIP-seq (Supplementary Figure 4 and Supplementary Table 2). These results suggest that the nCUT&Tag method could be applied for mapping active histone modifications with native nuclei.

Meanwhile, we performed H3K4me3-associated nCUT&Tag with crosslinked seedlings to compare with the fresh nCUT&Tag data (Supplementary Figure 5A). They showed a high correlation between the fixed and fresh nCUT&Tag (*r* = 0.89, Spearman’s correlation) (Supplementary Figure 5B). We detected 21,445 H3K4me3 peaks in crosslinked seedlings (Supplementary Table 1), among which 77% (16,468 peaks) were also detected in fresh seedlings by nCUT&Tag (Supplementary Figures 5C,D). Strikingly, about 10,175 peaks (∼38%) were exclusively detected in fresh tissues, suggesting that crosslinking might underpresent the detection of histone modifications. For cryopreserved seedlings, the crosslinked nuclei might need to be pre-opened with Hypotonic Buffer containing SDS, as described in CoBATCH and itChIP-seq (Ai et al., 2019; Wang et al., 2019), to capture much more signals.

H3K9me2, which shows a broad-peak profile in the rice genome, is a repressive histone mark associated with closely compacted heterochromatin (Zhao et al., 2020). To test whether nCUT&Tag can be used to characterize repressive chromatin features, we conducted another nCUT&Tag procedure with fresh rice seedlings to profile H3K9me2 histone modification.

The H3K9me2 nCUT&Tag showed a high correlation with our eChIP-seq data (*r* = 0.95, Spearman’s correlation) (Supplementary Figures 1C, 2B). The two biological replicates called 24,382 and 22,142 H3K9me2 peaks, respectively (Figure 4A and Supplementary Table 1). Among the 26,015 peaks identified by the two nCUT&Tag replicates, about 80% (20,646 peaks) were also detected by the eChIP-seq; 5369 peaks were detected by the fresh nCUT&Tag only, while 2244 were detected by eChIP-seq only (Figures 4C,E). The peak distribution showed a considerably consistency between the H3K9me2 nCUT&Tag and eChIP-seq results, with approximately 40% distributing at intergenic regions (Supplementary Figure 4 and Supplementary Table 2). We also compared the signal levels under the same sequencing depth between the nCUT&Tag and eChIP-seq libraries. The 8-M nCUT&Tag reads called 17,375 H3K9me2 peaks, which were almost as much as the 16-M reads from the eChIP-seq libraries (17,810 peaks) and even a little bit less than that from the 24-M eChIP-seq reads (18,954 peaks) (Supplementary Table 3). The results indicate that 8-M clean reads from nCUT&Tag provide comparable signals to the 16-M and even 24-M eChIP-seq reads.

Taken together, nCUT&Tag is a versatile method that can be used for global profiling of both active and repressive histone modifications in rice.

### nCUT&Tag for Efficient Chromatin Profiling With Low-Input Samples

**FIGURE 5:**
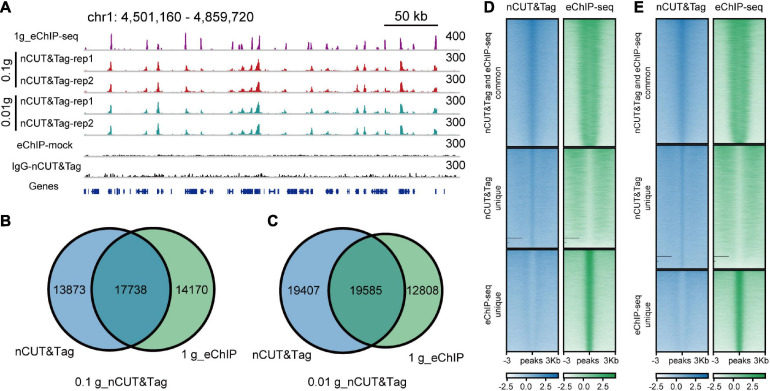
nCUT&Tag for chromatin landscape profiling with low-input samples. **(A)** Genome browser screenshot showing H3K4me3 nCUT&Tag data for low-input rice seedlings. The H3K4me3 eChIP-seq data were generated with 1-g crosslinked seedlings. **(B)** Venn diagram showing the overlap of H3K4me3 peaks detected by 0.1-g nCUT&Tag and crosslinked eChIP-seq. **(C)** Venn diagram showing the overlap of H3K4me3 peaks detected by 0.01-g nCUT&Tag and crosslinked eChIP-seq. **(D)** Comparison of H3K4me3 signals between 0.1-g nCUT&Tag and 1-g eChIP-seq. **(E)** Comparison of H3K4me3 signals between 0.01-g nCUT&Tag and 1-g eChIP-seq.

The standard ChIP-seq assay requires significant material (∼1 g). To test whether nCUT&Tag could profile histone modifications using low-input samples, we performed H3K4me3 nCUT&Tag with 0.1 and 0.01 g of crosslinked seedlings (Figure 5A). To avoid too much loss of nuclei in the centrifuge steps, here we use concanavalin A-coated magnetic beads for buffer exchange as an alternative strategy. The low-sample nCUT&Tag showed high correlations with both regular nCUT&Tag and eChIP-seq (the Spearman’s correlations varied from 0.89 to 0.93), and a high degree of consistency between the 0.1-g nCUT&Tag and 0.01-g nCUT&Tag (*r* = 1.00, Spearman’s correlation) (Supplementary Figures 1B, 2A). The 0.1- and 0.01-g nCUT&Tag detected 31,611 and 38,992 peaks, respectively. Among them, 17,738 (∼56% of 0.1-g nCUT&Tag) and 19,585 (∼50% of 0.01-g nCUT&Tag) peaks were detected by the 1-g eChIP-seq (Figures 5B-E). The signal showed lower enrichment at TSS than the regular nCUT&Tag and eChIP-seq (Supplementary Figure 3B). The peak distribution was also a little bit different from the regular nCUT&Tag and eChIP-seq, with less proportion of first-exon peaks and higher proportion of other-exon peaks (Supplementary Figure 4 and Supplementary Table 2). Overall, the low-input nCUT&Tag mapped many peaks commonly as detected by regular nCUT&Tag and eChIP-seq, but the signal was lower and the peak distribution was different. This means that it needs to be improved in the further.

### nCUT&Tag Is Scalable for Chromatin Profiling in Other Plant Species

**FIGURE 6:**
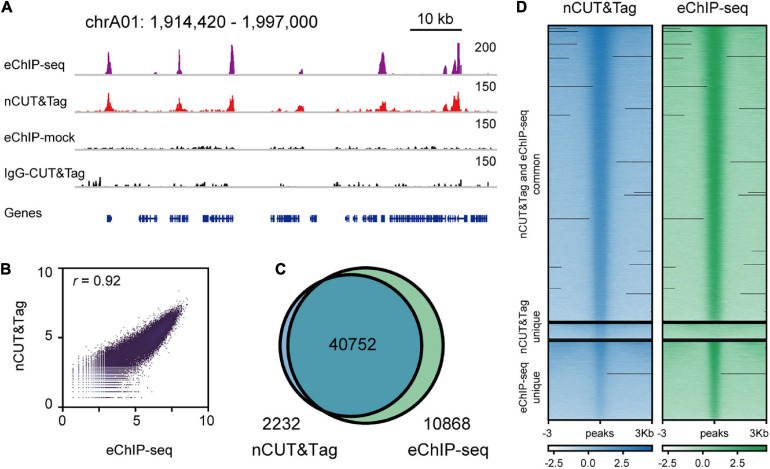
nCUT&Tag for fast chromatin profiling in *Brassica napus*. **(A)** Representative H3K4me3 landscapes across chrA01:1,914,420–1,996,975 of the *Brassica napus* genome generated by nCUT&Tag and eChIP-seq. **(B)** Scatter plots showing the Spearman’s correlation between the nCUT&Tag and eChIP-seq data. **(C)** Venn diagram showing the overlap of H3K4me3 peaks detected by nCUT&Tag and eChIP-seq. **(D)** Comparison of the H3K4me3 nCUT&Tag and eChIP-seq signals.

Enhanced ChIP-seq has been used to map high-quality reference epigenomes in rice and *B. napus* (Zhang et al., 2020; Zhao et al., 2020). To examine whether nCUT&Tag could be applied to other plant species, we generated the nCUT&Tag data of the H3K4me3 antibody with crosslinked leaves of the dicot rapeseed (*B. napus*) (Figure 6A). The rapeseed nCUT&Tag data showed a high correlation with the eChIP-seq (*r* = 0.92, Spearman’s correlation) (Figure 6B and Supplementary Figure 1D). The rapeseed nCUT&Tag totally identified 42,984 peaks and there were 10,868 peaks (∼20%) detected by eChIP-seq only (Figure 6C). However, there was slight signal enrichment in nCUT&Tag libraries at the 10,868 eChIP-seq unique peak regions (Figure 6D). As talked about in the rice fresh nCUT&Tag section, this may be due to the lower sequencing depth relative to the eChIP-seq libraries (Supplementary Table 4). The rapeseed nCUT&Tag showed similar signal profiles and peak distribution profiles (Supplementary Figures 3C, 4). The results indicate that nCUT&Tag can be used to study the chromatin landscapes in both monocots and dicots.

## Discussion

The standard ChIP-seq (Kaufmann et al., 2010) and eChIP-seq (Zhao et al., 2020) protocols for plants start with fresh tissue, followed by crosslinking, nuclei isolation, sonication, immunoprecipitation, reverse crosslinking, DNA extraction, and library preparation (including end repair, A-tailing, adaptor ligation, and PCR enrichment) (Table 1). The procedures are quite complex and require significant input samples and much time for completion. By contrast, nCUT&Tag is a crosslinking-free, sonication-free, immunoprecipitation-free strategy for *in situ* and *in vivo* detection of protein–DNA interactions (Table 1). It is a rapid and efficient protocol that all the procedures can be finished within 1 day with as little as 0.01 g of plant tissue.

**TABLE 1:** Comparison of nCUT&Tag with our previously reported eChIP-seq protocol.

	**nCUT&Tag**	**eChIP-seq**
Input samples	Fixed/fresh, low-input, scalable for single cells	Fixed, low-input
Sonication	No, sonication-free	Yes
Immunoprecipitation	No, immunoprecipitation-free	Protein G beads; low salt; high salt; LiCl buffer
Reverse crosslinking	Direct DNA purification for fresh tissue	Yes
Library preparation	PCR directly	End repair; A-tailing; adaptor ligation; PCR enrichment
Time from tissue to library	1 day	4 days

The sonication-based ChIP-seq assays might underpresent weak or indirect protein–DNA interactions, which might be disrupted during sonication (Fullwood and Ruan, 2009; Bi et al., 2017). For instance, the *Arabidopsis* NUP1 is a nuclear periphery-located protein that loosely interacts with repressive chromatin (Bi et al., 2017). With regular ChIP-seq procedures, the NUP1 peak signals cannot be detected. However, the RE-ChIP-seq (restriction enzyme-mediated ChIP-seq), in which the sonication-based chromatin fragmentation is replaced with restriction enzyme digestion, causes less disruption to protein–DNA interactions and observes signal enrichment of the loosely interacted chromatin positioned around the nuclear periphery (Bi et al., 2017). nCUT&Tag is a sonication-free method and detects ∼6000 unique peaks compared to eChIP-seq (Figures 3A,C-D). These peaks show narrower and weaker signals than those of commonly detected by nCUT&Tag and eChIP-seq (Figure 3D), suggesting that they are weak modification sites that are not efficiently preserved during sonication and thus cannot be detected in ChIP-seq assays. Therefore, the *in situ* method nCUT&Tag may have a broader spectrum in mapping *in vivo* protein–DNA interactions, especially for the weak or indirect interactions.

It is a key aspect of epigenomic study to map global chromatin features for understanding transcriptional regulation at single-cell levels. Currently, it is not realistic to perform sonication for a single cell. Therefore, the regular sonication-based ChIP-seq protocols are not suitable for single-cell epigenomic study. However, the PAT- or PGT-mediated sonication-free strategies such as CUT&Tag, ACT-seq, and CoBATCH can be used for single-cell, as well as high-throughput chromatin profiling (Carter et al., 2019; Kaya-Okur et al., 2019; Wang et al., 2019). Hence, nCUT&Tag may be scalable for high-throughput or single-nucleus profiling of histone marks in plants. Importantly, the PAT- or PGT-mediated chromatin immunocleavage strategies may greatly facilitate the development of single-cell ligation-free 3D genome mapping technologies (Ouyang et al., 2020).

Most recently, Liu et al. (2020) developed small-scale Tn5-assisted chromatin cleavage with sequencing (Stacc-seq) to map genome-wide occupancy of RNA polymerase II. The principle of Stacc-seq is similar to CUT&Tag, but the procedures are different. Stacc-seq starts with *in vitro* pre-incubation of antibody with PAT/PGT, followed by incubation of antibody-PAT/PGT complex with live cells (Liu et al., 2020). Compared to CUT&Tag, Stacc-seq adopts only one round of *in vivo* incubation, omitting many buffer-exchange steps. Hence, Stacc-seq can be used rapid profiling of histone marks and transcriptional factor occupancies with hundreds of cells. We believe that Stacc-seq, as well as nCUT&Tag, will be useful alternative methods of ChIP-seq.

## Conclusion

nCUT&Tag is a simple, rapid, and efficient method that is versatile for studying both active and repressive histone modifications across fresh and crosslinked plant tissues. It is a sonication-free and immunoprecipitation-free protocol that is scalable for single-nucleus chromatin profiling. Moreover, all the procedures in nCUT&Tag can be performed within 1 day with considerably low-input samples, paving a new avenue for rapid single-cell epigenomic studies in plants.

## Data Availability Statement

The datasets presented in this study can be found in online repositories. The names of the repository/repositories and accession number(s) can be found below: https://www.ncbi.nlm.nih.gov/, PRJNA671638.

## Author Contributions

WO and XL designed the experiments and wrote the manuscript. XL supervised the research. WO generated data with assistance from YP, QZ, and ZC. XZ, WO, and GL performed data analysis. All authors participated in data interpretation.

## Conflict of Interest

The authors declare that the research was conducted in the absence of any commercial or financial relationships that could be construed as a potential conflict of interest.

## Supplementary Material

Supplementary Figure 1 Correlation heatmaps for the nCUT&Tag and eChIP-seq data.

Supplementary Figure 2 Scatter plots for the nCUT&Tag and eChIP-seq data.

Supplementary Figure 3 Heatmaps for nCUT&Tag and eChIP-seq signals across gene body.

Supplementary Figure 4 Annotation of peaks for the H3K4me3 and H3K9me2 histone marks from nCUT&Tag and eChIP-seq data.

Supplementary Figure 5 Comparison of the chromatin landscape mapped by fresh nCUT&Tag and crosslinked nCUT&Tag.

Supplementary Table 1 Summary of the rice nCUT&Tag and eChIP-seq data.

Supplementary Table 2 Summary of the peak annotation.

Supplementary Table 3 Summary of peak numbers and FRiP values under various sequencing depths.

Supplementary Table 4 Summary of nCUT&Tag and eChIP-seq data from the *Brassica napus* young leaves.

## References

[B1] Ai S. Xiong H. Li C. C. Luo Y. Shi Q. Liu Y. (2019). Profiling chromatin states using single-cell itChIP-seq. *Nat. Cell Biol.* 21 1164–1172. 10.1038/s41556-019-0383-5 31481796

[B2] Bi X. Cheng Y. J. Hu B. Ma X. Wu R. Wang J. W. (2017). Nonrandom domain organization of the *Arabidopsis* genome at the nuclear periphery. *Genome Res.* 27 1162–1173. 10.1101/gr.215186.116 28385710 PMC5495068

[B3] Bolger A. M. Lohse M. Usadel B. (2014). Trimmomatic: a flexible trimmer for Illumina sequence data. *Bioinformatics* 30 2114–2120. 10.1093/bioinformatics/btu170 24695404 PMC4103590

[B4] Buenrostro J. D. Giresi P. G. Zaba L. C. Chang H. Y. Greenleaf W. J. (2013). Transposition of native chromatin for fast and sensitive epigenomic profiling of open chromatin, DNA-binding proteins and nucleosome position. *Nat. Methods* 10 1213–1218. 10.1038/nmeth.2688 24097267 PMC3959825

[B5] Carter B. Ku W. L. Kang J. Y. Hu G. Perrie J. Tang Q. (2019). Mapping histone modifications in low cell number and single cells using antibody-guided chromatin tagmentation (ACT-seq). *Nat. Commun.* 10:3747. 10.1038/s41467-019-11559-1 31431618 PMC6702168

[B6] Chalhoub B. Denoeud F. Liu S. Parkin I. A. P. Tang H. Wang X. (2014). Early allopolyploid evolution in the post-neolithic *Brassica napus* oilseed genome. *Science* 345 950–953. 10.1126/science.1253435 25146293

[B7] Fullwood M. J. Ruan Y. (2009). ChIP-based methods for the identification of long-range chromatin interactions. *J. Cell. Biochem.* 107 30–39. 10.1002/jcb.22116 19247990 PMC2748757

[B8] Heinz S. Benner C. Spann N. Bertolino E. Lin Y. C. Laslo P. (2010). Simple combinations of lineage-determining transcription factors prime cis-regulatory elements required for macrophage and B cell identities. *Mol. Cell* 38 576–589. 10.1016/j.molcel.2010.05.004 20513432 PMC2898526

[B9] Johnson D. S. Mortazavi A. Myers R. M. Wold B. (2007). Genome-wide mapping of in vivo protein-DNA interactions. *Science* 316 1497–1502. 10.1126/science.1141319 17540862

[B10] Kaufmann K. Muino J. M. Osteras M. Farinelli L. Krajewski P. Angenent G. C. (2010). Chromatin immunoprecipitation (ChIP) of plant transcription factors followed by sequencing (ChIP-SEQ) or hybridization to whole genome arrays (ChIP-CHIP). *Nat. Protoc.* 5 457–472. 10.1038/nprot.2009.244 20203663

[B11] Kaya-Okur H. S. Wu S. J. Codomo C. A. Pledger E. S. Bryson T. D. Henikoff J. G. (2019). CUT&Tag for efficient epigenomic profiling of small samples and single cells. *Nat. Commun.* 10:1930. 10.1038/s41467-019-09982-5 31036827 PMC6488672

[B12] Li H. Durbin R. (2009). Fast and accurate short read alignment with Burrows-Wheeler transform. *Bioinformatics* 25 1754–1760. 10.1093/bioinformatics/btp324 19451168 PMC2705234

[B13] Li H. Handsaker B. Wysoker A. Fennell T. Ruan J. Homer N. (2009). The sequence alignment/map format and SAMtools. *Bioinformatics* 25 2078–2079. 10.1093/bioinformatics/btp352 19505943 PMC2723002

[B14] Liu B. Xu Q. Wang Q. Feng S. Lai F. Wang P. (2020). The landscape of RNA Pol II binding reveals a stepwise transition during ZGA. *Nature* 587 139–144. 10.1038/s41586-020-2847-y 33116310

[B15] Ouyang W. Xiong D. Li G. Li X. (2020). Unraveling the 3D genome architecture in plants: present and future. *Mol. Plant* 13 1676–1693. 10.1016/j.molp.2020.10.002 33065269

[B16] Ramirez F. Dundar F. Diehl S. Gruning B. A. Manke T. (2014). deepTools: a flexible platform for exploring deep-sequencing data. *Nucleic Acids Res.* 42 W187–W191. 10.1093/nar/gku365 24799436 PMC4086134

[B17] Skene P. J. Henikoff J. G. Henikoff S. (2018). Targeted in situ genome-wide profiling with high efficiency for low cell numbers. *Nat. Protoc.* 13 1006–1019. 10.1038/nprot.2018.015 29651053

[B18] Sun Y. Dong L. Zhang Y. Lin D. Xu W. Ke C. (2020). 3D genome architecture coordinates trans and cis regulation of differentially expressed ear and tassel genes in maize. *Genome Biol.* 21:143. 10.1186/s13059-020-02063-7 32546248 PMC7296987

[B19] Tao X. Feng S. Zhao T. Guan X. (2020). Efficient chromatin profiling of H3K4me3 modification in cotton using CUT&Tag. *Plant Methods* 16:120. 10.1186/s13007-020-00664-8 32884577 PMC7460760

[B20] Wang Q. Xiong H. Ai S. Yu X. Liu Y. Zhang J. (2019). CoBATCH for high-throughput single-cell epigenomic profiling. *Mol. Cell* 76 206.e7–216.e7. 10.1016/j.molcel.2019.07.015 31471188

[B21] Zhang J. Chen L. L. Xing F. Kudrna D. A. Yao W. Copetti D. (2016). Extensive sequence divergence between the reference genomes of two elite indica rice varieties Zhenshan 97 and Minghui 63. *Proc. Natl. Acad. Sci. U.S.A.* 113 E5163–E5171. 10.1073/pnas.1611012113 27535938 PMC5024649

[B22] Zhang Q. Guan P. Zhao L. Ma M. Xie L. Yue L. (2020). Asymmetric epigenome maps of subgenomes reveal imbalanced transcription and distinct evolutionary trends in *Brassica napus*. *Mol. Plant* [Epub ahead of print] 10.1016/j.molp.2020.12.020 33387675

[B23] Zhang Y. Liu T. Meyer C. A. Eeckhoute J. Johnson D. S. Bernstein B. E. (2008). Model-based analysis of ChIP-Seq (MACS). *Genome Biol.* 9:R137. 10.1186/gb-2008-9-9-r137 18798982 PMC2592715

[B24] Zhao L. Xie L. Zhang Q. Ouyang W. Deng L. Guan P. (2020). Integrative analysis of reference epigenomes in 20 rice varieties. *Nat. Commun.* 11:2658. 10.1038/s41467-020-16457-5 32461553 PMC7253419

[B25] Zheng X. Y. Gehring M. (2019). Low-input chromatin profiling in *Arabidopsis* endosperm using CUT&RUN. *Plant Reprod.* 32 63–75. 10.1007/s00497-018-00358-1 30719569

