# Supplementary material for: Rapid and Low-Input Profiling of Histone Marks in Plants Using Nucleus CUT&Tag
Source: Front Plant Sci. 2021 Apr 12;12:634679. doi: 10.3389/fpls.2021.634679 (PMC8072009; doi:10.3389/fpls.2021.634679)
Supplement: Supplementary Figure 1 — Correlation heatmaps for the nCUT&Tag and eChIP-seq data. [file Data_Sheet_1.docx]

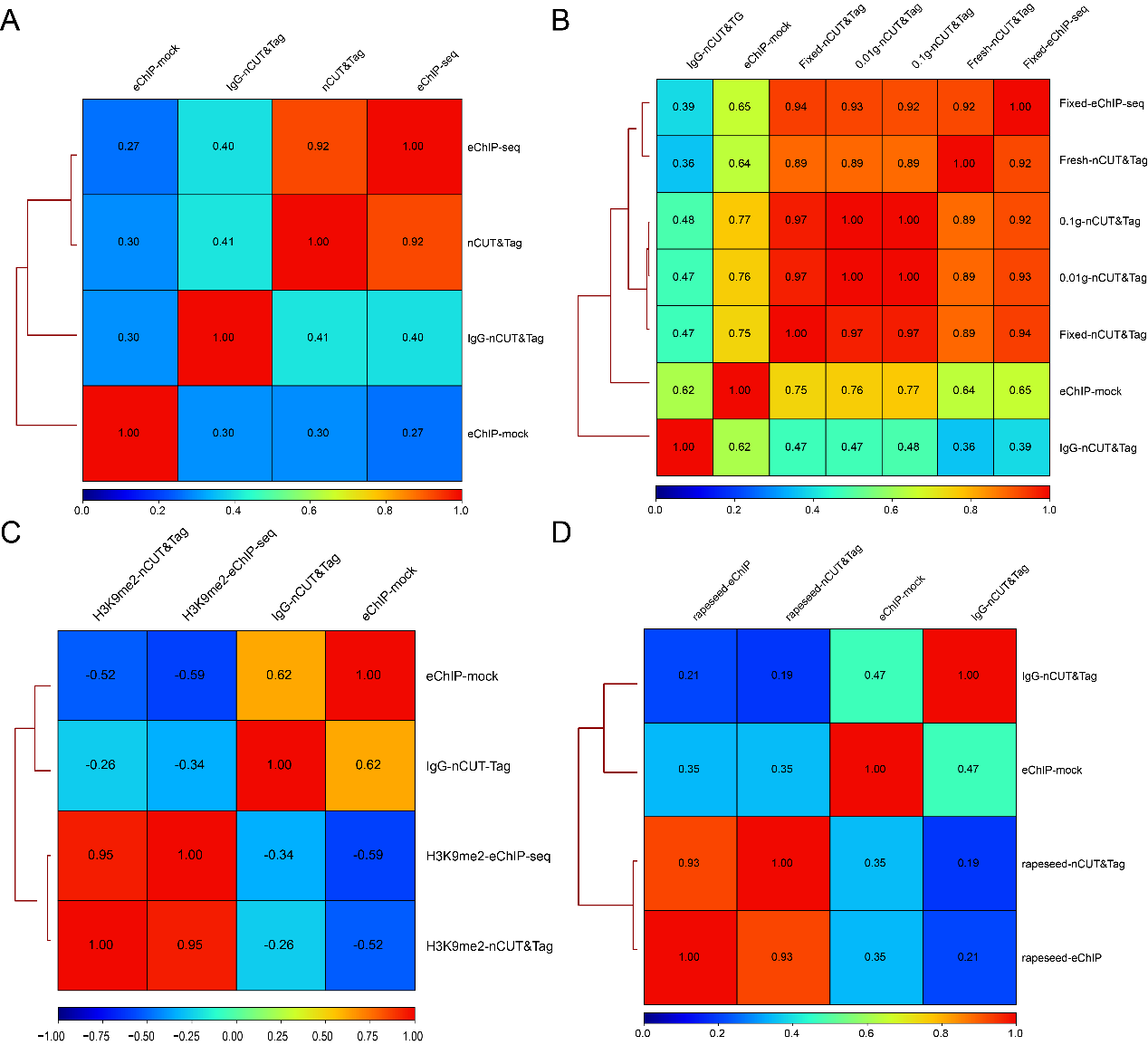


**SUPPLEMENTARY FIGURE S1 |** Correlation heatmaps for the nCUT&Tag and eChIP-seq data. (**A**) Correlation heatmap for the crosslinked nCUT&Tag and eChIP-seq data from rice panicles. eChIP-mock was performed following our eChIP-seq protocol. The IgG-nCUT&Tag was conducted in parallel with the H3K4me3 nCUT&Tag. (**B**) Correlation heatmap for the fresh H3K4me3 nCUT&Tag, fixed nCUT&Tag, 0.1-g nCUT&Tag, 0.01-g nCUT&Tag, and 1-g fixed eChIP-seq data from rice seedlings. (**C**) Correlation heatmap for the fresh H3K9me2 nCUT&Tag and crosslinked H3K9me2 eChIP-seq data from rice seedlings. (**D**) Correlation heatmap for the fresh H3K4me3 nCUT&Tag and crosslinked eChIP-seq data from Brassica napus young leaves.


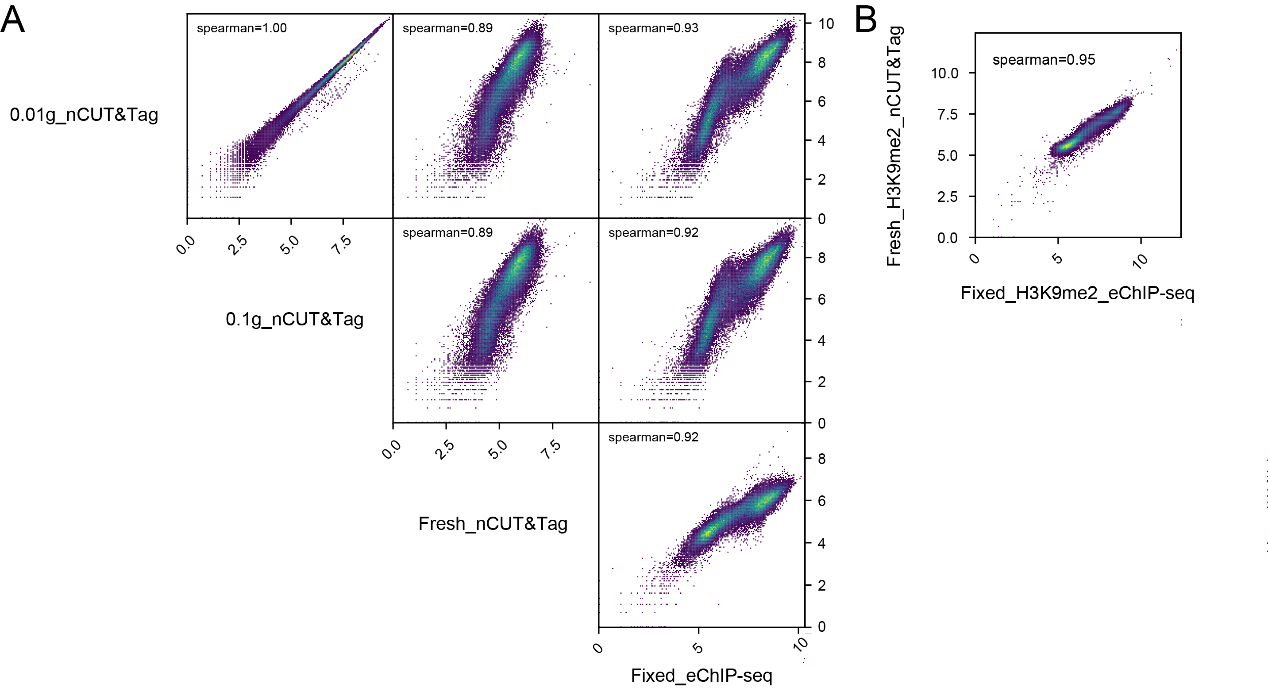


**SUPPLEMENTARY FIGURE S2 |** Scatter plots for the nCUT&Tag and eChIP-seq data. (**A**) Scatter plots for the fresh H3K4me3 nCUT&Tag, 0.1-g nCUT&Tag, 0.01-g nCUT&Tag, and 1-g fixed eChIP-seq data from rice seedlings. (**B**) Scatter plot for the fresh H3K9me2 nCUT&Tag and crosslinked eChIP-seq data from rice seedlings.


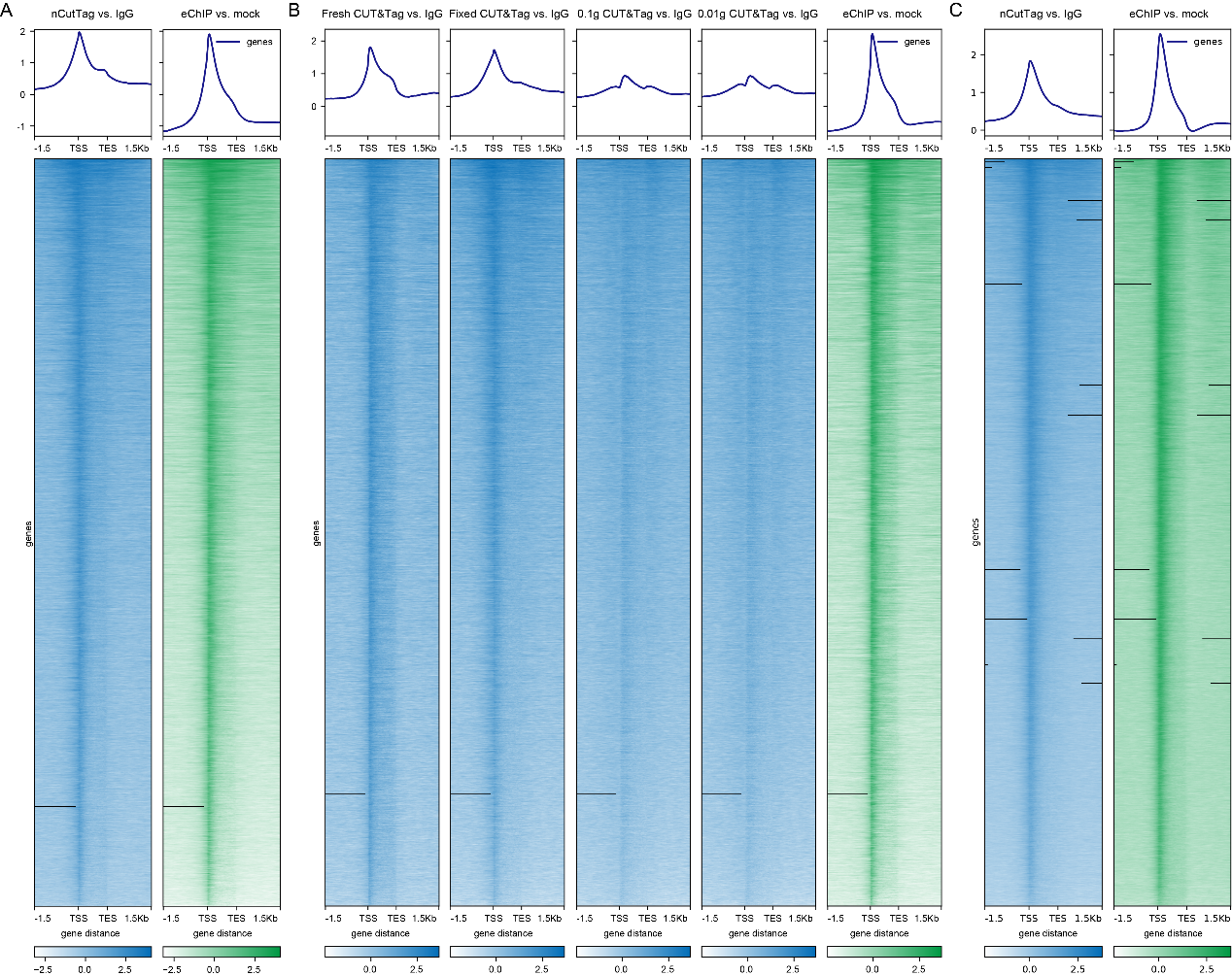


**SUPPLEMENTARY FIGURE S3 |** Heatmaps for nCUT&Tag and eChIP-seq signals across gene body. (**A**) Heatmap for the crosslinked panicle nCUT&Tag and eChIP-seq signals across gene body. (**B**) Heatmaps for the rice fresh seedling H3K4me3-nCUT&Tag, fixed nCUT&Tag, 0.1-g nCUT&Tag, 0.01-g nCUT&Tag, and 1-g fixed eChIP-seq signals across gene body. (**C**) Heatmap for the fresh H3K4me3 nCUT&Tag and crosslinked eChIP-seq signals across gene body from Brassica napus young leaves.


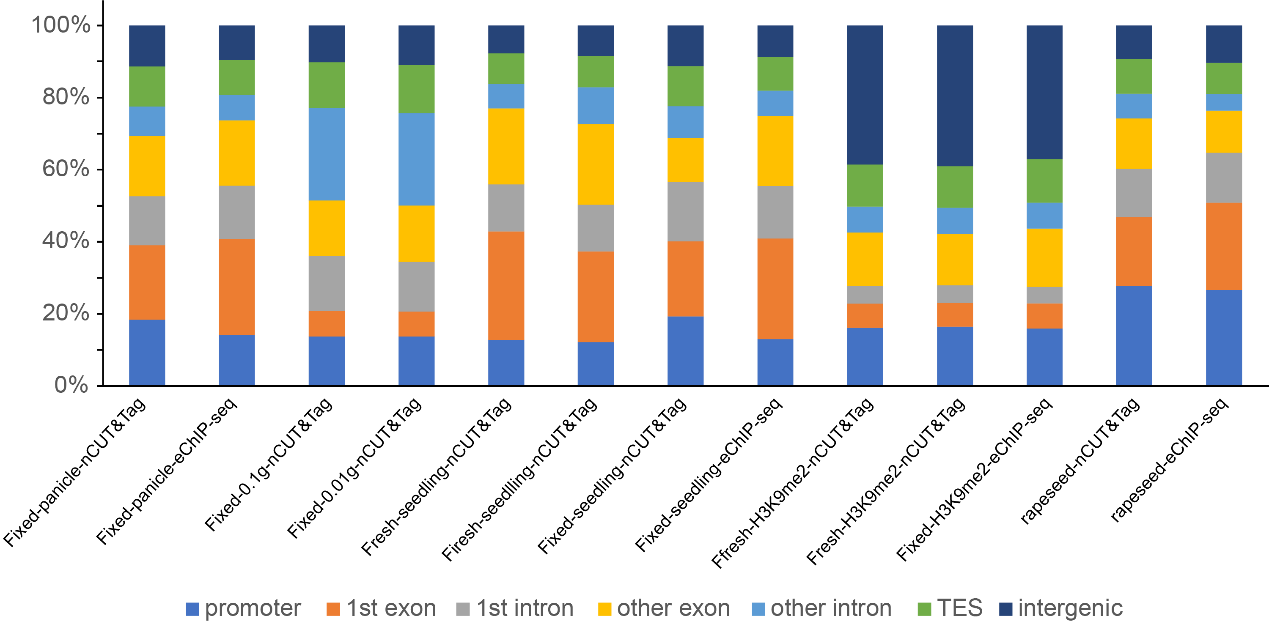


**SUPPLEMENTARY FIGURE S4 |** Annotation of peaks for the H3K4me3 and H3K9me2 histone marks from nCUT&Tag and eChIP-seq data. The peak distribution regions were annotated as promoters, first exons, first introns, other exons, other introns, transcription end sites, and intergenic regions. The y-axis showed the proportions for different annotated peaks from each library.


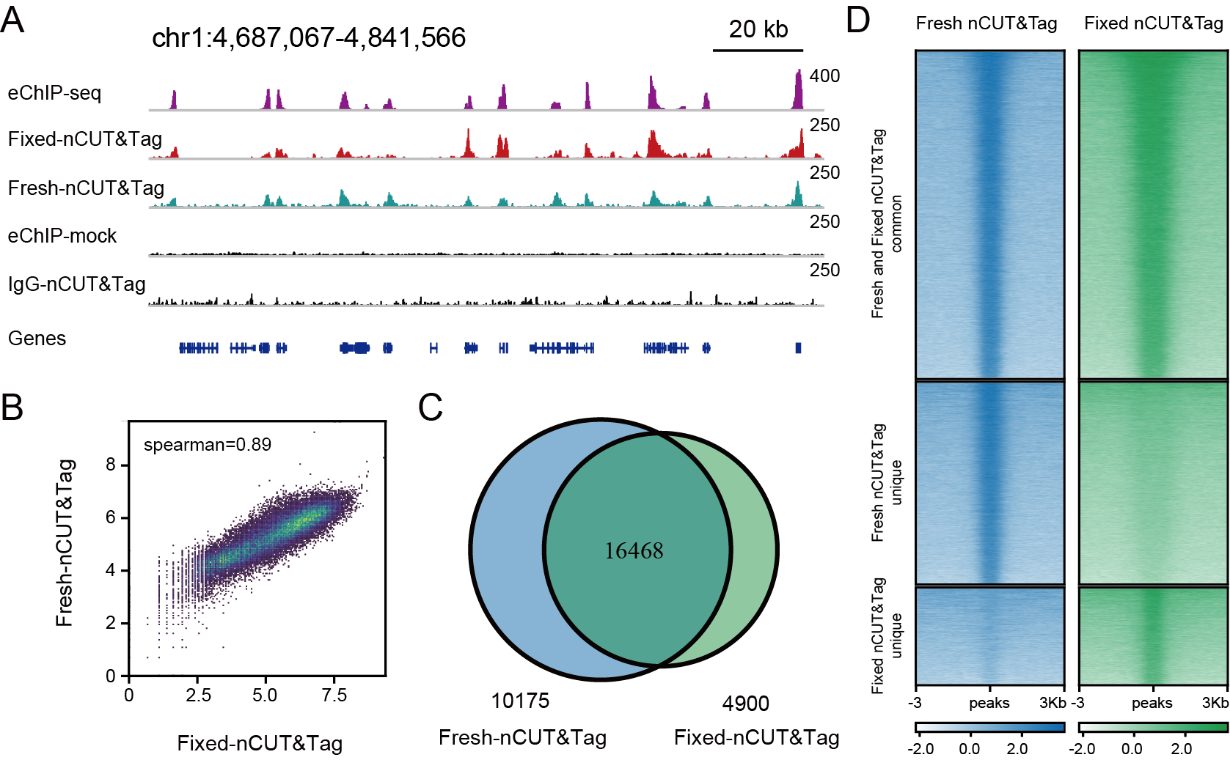


**SUPPLEMENTARY FIGURE S5 |** Comparison of the chromatin landscape mapped by fresh nCUT&Tag and fixed nCUT&Tag. **(A)** Representative H3K4me3 landscapes across chr1:4,687,067–4,841,566 of the rice genome generated by nCUT&Tag and eChIP-seq. **(B)** Scatter plots showing the Spearman’s correlation between the fresh nCUT&Tag and crosslinked nCUT&Tag. **(C)** Venn diagram showing the overlap of H3K4me3 peaks detected by fresh and fixed nCUT&Tag. **(D)** Comparison of the H3K4me3 signals between fresh and fixed nCUT&Tag.

**SUPPLEMENTARY TABLE S1 |** Summary of the rice nCUT&Tag and eChIP-seq data.

| Antibody | tissue | method | Library ID | Raw reads | Clean Reads | Mapped reads | Mapping rate | Peak No. | FRiP |
| --- | --- | --- | --- | --- | --- | --- | --- | --- | --- |
| H3K4me3 | Panicle-X | nCUT&Tag | Fixed-panicle-nCUT&Tag-rep1 | 34,603,340 | 33,613,059 | 33,168,247 | 98.68% | 31,436 | 58.12% |
|  | Panicle-X | nCUT&Tag | Fixed-panicle-nCUT&Tag-rep2 | 33,449,460 | 31,950,265 | 31,647,204 | 99.05% | 27,857 | 56.38% |
|  | Panicle-X | eChIP-seq | Fixed-panicle-eChIP-rep1 | 50,708,728 | 50,114,085 | 49,148,905 | 98.07% | 29,730 | 72.75% |
|  | Panicle-X | eChIP-seq | Fixed-panicle-eChIP-rep2 | 23,568,468 | 23,262,895 | 22,805,481 | 98.03% | 27,782 | 71.32% |
|  | Seedling-N | nCUT&Tag | Fresh-K43-nCUT&Tag-rep1 | 9,787,952 | 8,489,494 | 8,426,964 | 99.20% | 21,203 | 30.78% |
|  | Seedling-N | nCUT&Tag | Fresh-K43-nCUT&Tag-rep2 | 5,806,154 | 5,059,652 | 5,032,792 | 99.38% | 23,545 | 44.32% |
|  | Seedling-X | nCUT&Tag | Fixed-K43-nCUT&tag | 19,232,223 | 17,021,143 | 33,909,484 | 99.52% | 21,445 | 45.33% |
|  | Seedling-X | nCUT&Tag | Fixed-K43-0.1g-nCUT&Tag-rep1 | 42,374,060 | 39,565,554 | 39,427,392 | 99.61% | 28,993 | 36.04% |
|  | Seedling-X | nCUT&Tag | Fixed-K43-0.1g-nCUT&Tag-rep2 | 26,324,796 | 24,516,498 | 24,429,652 | 99.61% | 24,892 | 32.55% |
|  | Seedling-X | nCUT&Tag | Fixed-K43-0.01g-nCUT&Tag-rep1 | 60,713,510 | 56,721,234 | 56,560,134 | 99.69% | 35,084 | 40.31% |
|  | Seedling-X | nCUT&Tag | Fixed-K43-0.01g-nCUT&Tag-rep2 | 46,494,804 | 43,740,888 | 43,612,517 | 99.68% | 33,069 | 38.56% |
|  | Seedling-X | eChIP-seq | Fixed-K43-eChIP-rep1 | 52,808,410 | 49,380,979 | 47,157,701 | 96.05% | 27,516 | 69.30% |
|  | Seedling-X | eChIP-seq | Fixed-K43-eChIP-rep2 | 51,996,256 | 47,818,942 | 46,346,157 | 96.92% | 30,230 | 73.57% |
| H3K9me2 | Seedling-N | nCUT&Tag | Fresh-K92-nCUT&Tag-rep1 | 32,423,802 | 29,007,062 | 28,726,207 | 98.90% | 24,382 | 46.34% |
|  | Seedling-N | nCUT&Tag | Fresh-K92-nCUT&Tag-rep2 | 22,376,124 | 20,049,814 | 19,912,452 | 99.18% | 22,142 | 50.50% |
|  | Seedling-X | eChIP-seq | Fixed-K92-eChIP-rep1 | 67,743,714 | 61,472,015 | 60,988,077 | 99.21% | 19,589 | 53.12% |
|  | Seedling-X | eChIP-seq | Fixed-K92-eChIP-rep2 | 36,291,684 | 34,669,137 | 31,375,536 | 90.50% | 20,666 | 45.81% |
| Mock/IgG | Panicle-X | ChIP-mock | Fixed-panicle-input | 42,403,920 | 40,547,999 | 35,504,951 | 87.56% | 1,733 | 3.93% |
|  | Seedling-X | ChIP-mock | Fixed-seedling-input | 18,309,092 | 17,569,054 | 16,481,481 | 93.81% | 294 | 9.34% |
|  | Seedling-N | nCUT&Tag | Fresh-seedling-IgG-nCUT&Tag | 11,871,678 | 10,266,632 | 9,626,222 | 92.92% | 135 | 12.73% |

FRiP, Fraction of Reads in Peaks.

**SUPPLEMENTARY TABLE S2 |** Summary of the peak annotation.

| Library ID | Promoter | 1^st^ exon | 1^st^ intron | Other exons | Other introns | TES | Intergenic |
| --- | --- | --- | --- | --- | --- | --- | --- |
| Fixed-panicle-nCUT&Tag | 5805 (18.44%) | 6496 (20.63%) | 4283 (13.60%) | 5245 (16.66%) | 2582 (8.20%) | 3508 (11.14%) | 3565 (11.32%) |
| Fixed-panicle-eChIP-seq | 4207 (14.13%) | 7927 (26.63%) | 4422 (14.86%) | 5396 (18.13%) | 2120 (7.12%) | 2843 (9.55%) | 2852 (9.58%) |
| Fixed-K43-0.1g-nCUT&Tag | 4327 (13.69%) | 2237 (7.08%) | 4860 (15.37%) | 4843 (15.32%) | 8109 (25.65%) | 4019 (12.71%) | 3217 (10.18%) |
| Fixed-K43-0.01g-nCUT&Tag | 5348 (13.71%) | 2707 (6.94%) | 5389 (13.82%) | 6091 (15.62%) | 9997 (25.63%) | 5192 (13.31%) | 4275 (10.96%) |
| Fresh-K43-nCUT&Tag-rep1 | 3410 (12.80%) | 8005 (30.04%) | 3480 (13.06%) | 5617 (21.08%) | 1823 (6.84%) | 2274 (8.53%) | 2036 (7.64%) |
| Fresh-K43-nCUT&Tag-rep2 | 4140 (12.18%) | 8550 (25.16%) | 4427 (13.03%) | 7568 (22.27%) | 3487 (10.26%) | 2922 (8.60%) | 2887 (8.50%) |
| Fixed-K43-nCUT&tag | 4118 (19.27%) | 4454 (20.84%) | 3535 (16.54%) | 2598 (12.16%) | 1891 (8.85%) | 2379 (11.13%) | 2398 (11.22%) |
| Fixed-K43-eChIP | 3906 (12.94%) | 8451 (27.99%) | 4385 (14.52%) | 5894 (19.52%) | 2097 (6.95%) | 2826 (9.36%) | 2631 (8.71%) |
| Fresh-K92-nCUT&Tag-rep1 | 4184 (16.08%) | 1750 (6.73%) | 1249 (4.80%) | 3894 (14.97%) | 1887 (7.25%) | 3017 (11.60%) | 10035 (38.57%) |
| Fresh-K92-nCUT&Tag-rep2 | 4710 (16.40%) | 1929 (6.72%) | 1386 (4.83%) | 4089 (14.24%) | 2087 (7.27%) | 3319 (11.56%) | 11198 (38.99%) |
| Fixed-K92-eChIP | 3369 (15.89%) | 1500 (7.08%) | 951 (4.49%) | 3434 (16.20%) | 1527 (7.20%) | 2561 (12.08%) | 7854 (37.05%) |
| Rapeseed-nCUT&Tag | 11897 (27.68%) | 8246 (19.18%) | 5760 (13.40%) | 6017 (14.00%) | 2938 (6.84%) | 4135 (9.62%) | 3991 (9.28%) |
| Rapeseed-eChIP-seq | 13850 (26.54%) | 12716 (24.37%) | 7226 (11.63%) | 6068 (11.63%) | 2408 (4.61%) | 4511 (8.65%) | 5400 (10.35%) |

TES, transcription end site.

**SUPPLEMENTARY TABLE S3 |** Summary of peak numbers and FRiP values under various sequencing depth.

| library | 1 M | 2 M | 4 M | 8 M | 16 M | 24 M |
| --- | --- | --- | --- | --- | --- | --- |
| Fixed-panicle-nCUT&Tag | 9,232 (22.67%) | 16,323 (35.24%) | 22,020 (44.93%) | 27,043 (52.13%) | 30,178 (56.81%) |  |
| Fixed-panicle-eChIP-seq | 16,452 (49.20%) | 20,554 (58.60%) | 23,053 (63.75%) | 26,109 (68.01%) | 27,659 (70.39%) | 28,630 (71.57%) |
| Fresh-K92-nCUT&Tag | 88 (0.14%) | 1,584 (1.97%) | 6,934 (8.38%) | 17,375 (23.77%) | 23,421 (38.94%) |  |
| Fixed-K92-eChIP | 70 (0.13%) | 808 (1.17%) | 3,911 (5.91%) | 11,478 (19.27%) | 17,810 (36.69%) | 18,954 (44.92%) |
| eChIP-mock | 93 (2.28%) | 106 (2.50%) | 179 (2.70%) | 403 (3.02%) | 956 (3.48%) |  |
| IgG-nCUT&Tag | 84 (11.75%) | 101 (12.19%) |  |  |  |  |

FRiP, Fraction of Reads in Peaks.

| Antibody | tissue | method | Library ID | Raw reads | Clean Reads | Mapped reads | Mapping rate | Peak No. | FRiP |
| --- | --- | --- | --- | --- | --- | --- | --- | --- | --- |
| H3K4me3 | Seedling-X | nCUT&Tag | Rapeseed-nCUT&Tag-rep1 | 13,007,834 | 11,298,468 | 11,321,479 | 99.08% | 42,035 | 50.97% |
|  | Seedling-X | nCUT&Tag | Rapeseed-nCUT&Tag-rep2 | 4,879,026 | 3,779,804 | 3,813,285 | 99.18% | 25,696 | 32.74% |
|  | Seedling-X | eChIP-seq | Rapeseed-eChIP | 38,283,320 | 37,368,352 | 35,482,308 | 94.95% | 52,251 | 63.91% |
| Mock/IgG | Seedling-X | ChIP-mock | Rapeseed-seedling-input | 10,791,813 | 10,721,830 | 10,668,037 | 99.50% | 249 | 3.00% |
|  | Seedling-X | nCUT&Tag | Rapeseed-seedling-IgG-nCUT&Tag | 9,551,478 | 8,438,216 | 7,958,055 | 93.02% | 700 | 4.90% |

**SUPPLEMENTARY TABLE S4 |** Summary of nCUT&Tag and eChIP-seq data from the *Brassica napus* young leaves.

FRiP, Fraction of Reads in Peaks.
